# Evaluation of the effect of endometrial scratch by hysteroscopic scissors on frozen embryo transfer outcomes: A historical cohort study

**DOI:** 10.18502/ijrm.v21i9.14400

**Published:** 2023-10-30

**Authors:** Sara Saedi, Amirhossein Tayebi, Maedeh Ghorbani Kahrizsangi, Fatemeh Jalalinezhad, Aryan Ayati, Alireza Hadizadeh, Bita Badehnoosh, Atousa Karimi

**Affiliations:** ^1^Reproductive Biotechnology Research Center, Avicenna Research Institute, ACECR, Tehran, Iran.; ^2^Cardiovascular Research Center, Alborz University of Medical Sciences, Karaj, Iran.; ^3^Research Center for Advanced Technologies in Cardiovascular Medicine, Cardiovascular Diseases Research Institute, Tehran University of Medical Sciences, Tehran, Iran.

**Keywords:** Hysteroscopy, Infertility, Pregnancy rate, Live birth.

## Abstract

**Background:**

Endometrial scratch (ES) has been suggested to improve assisted reproductive techniques success rates by investigating implantation failure.

**Objective:**

In this study, we evaluated the effect of ES on the outcomes of frozen embryo transfer (FET) in women with at least 2 failed embryo transfer cycles.

**Materials and Methods:**

In this historical cohort study, medical data of 236 infertile women who underwent in-vitro fertilization-FET at Ebne-sina Infertility Center, Tehran, Iran, from January 2015-December 2021 was extracted from their medical records. Based on having ES before FET, they were assigned to either the scratch (n = 118) or the no-scratch group (n = 118). We compared these groups regarding pregnancy rates and outcomes.

**Results:**

The demographic characteristics were similar in both groups regarding weight, body mass index, the number of previous embryo transfers, and the duration of infertility. However, the scratch group had a slightly higher mean age (32.31 vs. 29.96 yr, p < 0.001). No statistically significant difference was observed between groups regarding pregnancy rate (p = 0.89). No significant association was observed between scratch, infertility duration, the number of previous FET attempts, and the likelihood of pregnancy in a logistic regression model. No major complications were observed.

**Conclusion:**

Hysteroscopic endometrial scratching with scissors probably has no effect on FET outcomes, including pregnancy or live birth rates.

## 1. Introduction

Based on archived reports from the Centers for Disease Control and Prevention, the number of performed assisted reproductive techniques (ART) cycles has doubled from 2009-2018 (1). Despite the advancements in ART over the past decades, the pregnancy rate per embryo transfer remains below 35% (2). The growing demand for assisted reproduction underscores the importance of improving the ART success rate. An essential aspect of this pursuit is understanding endometrial tolerance during embryo implantation, a critical holdup in achieving a successful ART (3, 4). In response to this need, a recent international consensus has emphasized the need for investigating implantation failure as a top research priority in medically assisted reproduction (5).

A study of endometrial gap junction proteins found that most women who failed in-vitro fertilization (IVF) and underwent repetitive endometrial sampling conceived in the following cycle. Building on this observation, they studied 134 cases with at least one IVF failure and reported that endometrial injury (pipelle biopsy) could double the IVF success rate (6). Since the publication of this study, endometrial scratch has gained considerable traction, prompting substantial debate within the scientific community.

Frozen embryo transfer (FET) is a widely employed ART procedure that transfers cryopreserved embryos into the uterus. 2 decades following the inception of the first ES procedure, its advantages remain controversial.

While most studies worked on the effects of ES on fresh embryo transfer, we conducted this study to evaluate the effect of hysteroscopy and ES on the outcomes of FET in women with at least 2 failed embryo transfer cycles.

## 2. Materials and Methods

### Participants and setting

This historical cohort study assessed on medical data of 236 women who underwent intracytoplasmic sperm injection at Ebne-sina Infertility Center, Tehran, Iran, from January 2015-December 2021. The demographic characteristics, infertility data, and pregnancy follow-ups were retrieved from medical records utilizing study checklists. Using the Excel random list function, we randomly selected 420 cases from the database. Out of these, 184 women did not meet our inclusion criteria. The remaining 236 women had undergone the same medical treatments and FET cycle.

### Inclusion criteria

1. Infertile women with a history of 2 unsuccessful embryo implantations (infertility was defined based on the history of at least 1 yr of regular and unprotected intercourse without a pregnancy).

2. Underwent FET at Ebne-sina Infertility Center, Tehran, Iran, between January 2015 and December 2021.

3. Body mass index (BMI) < 30 during the procedure.

4. Age < 39 yr at the time of the procedure.

### Exclusion criteria

1. Structural abnormalities of the uterus include congenital (bicornuate, unicornuate, or uterine septum) or acquired disorders (submucosal myomas, Asherman's syndrome).

2. Uncontrolled chronic medical disease (diabetes, hypertension, toxoplasmosis, hypo or hyperthyroidism, BMI ≥ 30).

3. History of cancer or autoimmune diseases (multiple sclerosis, Lupus, rheumatoid arthritis, thyroid peroxidase antibody > 3 times the normal), thrombophilia or acquired coagulation disorders, immuno-suppressor medications, and abnormal karyotypes.

4. History of azoospermia or receiving donor oocytes.

### Variables and measurements

Participants' data were collected from each couple's personalized infertility files according to the study checklist, including age, weight, BMI, infertility duration, infertility causes, pre- and post-FET treatments, ultrasound findings, and outcomes. The duration of infertility was determined based on the history of consistent and unprotected intercourse without resulting in a pregnancy. The infertility cause was evaluated by infertility fellows who examined the cases and categorized the causes as male infertility, female infertility (ovulatory dysfunction, tubal pathologies, and others), or both. Depending on whether the individuals had ES before FET, they were assigned to either the scratch (n = 118) or the no-scratch group (n = 118).

### Hysteroscopy and endometrial scratch

The following method is used at the Ebne-sina Infertility Center for endometrial scratch. Women in the scratch group had undergone hysteroscopic ES using scissors one cycle before FET, between the 15 ${}^{\text{th}}$ and 20 ${}^{\text{th}}$ day of their menstrual cycle during the luteal phase. The procedure was performed by an infertility specialist using hysteroscopy. Under general anesthesia and in a lithotomy position, a 4 mm lens was introduced to the uterus through the cervix. The uterine cavity was expanded by injecting a low-density liquid (normal saline) to visualize the uterine walls better. Finally, fine scratches on the endometrium were performed by hysteroscopic scissors.

On the 1 ${}^{\text{st}}$ -3 ${}^{\text{rd}}$ day of the cycle, all participants underwent an ultrasound to assess the thickness of the endometrium. The pregnancy beta-human chorionic gonadotropin test was performed 14 and 16 days after the FET.

### Outcomes

A comprehensive comparison of the mentioned groups was conducted, focusing on both pregnancy rates and associated outcomes.

### Ethical considerations

A written informed consent was obtained from each individual at the time of admission for possible usage of their data for research purposes. This project was approved by the Ethics Committee in Research at Avicenna Research Institute, Tehran, Iran (Code: IR.ACER.AVICENNA.REC.1401.002).

### Statistical analysis

The study population was split into 2 groups, based on ES. Categorical data were depicted as frequencies (in percentages) and assessed through a Chi-square test, while continuous data were presented as either mean ± standard deviation or median (interquartile range), and their comparison was carried out using Pearson Chi-square and Fisher's exact test. For multivariate Cox regression analysis, variables with a significance level of p < 0.2 were integrated into the regression model using the backward selection method. All statistical computations were executed using SPSS version 22.0 developed by IBM in Armonk, New York.

## 3. Results

Of the 420 initially selected cases, 236 women were included in the study. Based on receiving ES prior to IVF, 118 cases were allocated to the ES group, and the rest (n = 118) to the control group (Table I). Participants mean age was 31.13 ± 4.51 yr, with a mean BMI of 24.88 ± 3.12 Kg/m^2^. Furthermore, they had an average infertility duration of 6.23 ± 3.65 yr, slightly higher in those who received scratching (p = 0.09). Participants had previously undergone embryo transfers 2-11 times with a median (interquartile range) of 3 (2, 4) (Table I).

Female infertility was reported in 94.5% and male infertility in 58.1% of couples. Regarding female infertility, most cases were due to ovulatory dysfunction, and tubal pathologies were reported in only 3.0% of cases. No significance was observed between the 2 groups regarding the cause of infertility (Table I).

After the follow-up period of the participants, pregnancy was noted in 75 (31.8%), 38 (32.2%) in the scratch group, and 37 (31.4%) women in the control group, indicating no statistically significant difference (p = 0.89). Of the pregnancies, 9 (12.0%) resulted in an abortion and 66 (88.0%) in a live birth. The live birth rate was 34 (91.9%) in the control group and 32 (84.2%) in the scratch group. No significant difference was observed regarding the pregnancy outcome among the 2 groups (p = 0.48) (Table I). Also, none of the cases experienced any in-center complications, including excessive pain, vaginal bleeding, surgical site infection, and uterine perforation.

Binomial logistic regression was performed to ascertain the effects of scratch, infertility duration, and the number of previous FET attempts on the likelihood of pregnancy. The model was statistically nonsignificant (χ2 = 2.546, p = 0.49), showing no significant association (Table II).

**Table 1:** Comparison of characteristics and outcomes of infertile women in groups (n = 118/each)


**Variable**		**No-scratch**	**Scratch**	**P-value**
Age (yr)*		29.96 ± 4.31	32.31 ± 4.41	< 0.01
Weight (kg)*		65.95 ± 9.36	65.40 ± 9.34	0.65
Body mass index (kg/m^2^)*		25.22 ± 3.07	24.88 ± 3.12	0.40
Infertility duration (yr)*		5.80 ± 3.60	6.64 ± 3.67	0.09
Number of previous embryo transfers**		3 (2, 5)	3 (2, 4)	0.07
**Cause of infertility**				
	Male infertility***	74 (62.7)	63 (53.4)	0.15
	Female infertility***	109 (92.4)	114 (96.6)	0.15
	Ovulatory dysfunction***	42 (35.6)	47 (39.8)	0.50
**Cause of infertility**				
	Tubal pathology****	5 (4.2)	2 (1.7)	0.25
	Others***	62 (52.5)	66 (55.9)	0.60
	Both***	66 (55.9)	58 (49.2)	0.30
**Outcomes**				
	Pregnancy***	37 (31.4)	38 (32.2)	0.89
	Abortion****	3 (8.1)	6 (15.8)	0.48
	Live birth***	34 (91.9)	32 (84.2)	0.48
*Data presented as Mean ± SD. Paired sample *t *test, **Data presented as median (Interquartile range), Mann-Whitney U test, ***Data presented as n (%), Pearson Chi-square, ****Data presented as n (%), Fisher's Exact test				

**Table 2:** Binomial logistic regression predicting the likelihood of pregnancy


**Variables **	**B (intercept)**	**Standard error**	**Wald Chi-square**	**P-value**	**Odds ratio**
Endometrial scratch*	0.076	0.306	0.062	0.80	1.079
**Age**	-0.013	0.034	0.154	0.69	0.987
**Body mass index**	-0.066	0.049	1.782	0.18	0.936
**Infertility duration**	0.023	0.044	0.270	0.60	1.023
**Number of previous** **embryo transfers**	-0.088	0.101	0.764	0.38	0.916
**Constant**	1.453	1.699	0.731	0.39	4.276
*Categorica					

## 4. Discussion

This retrospective cohort study evaluated the impact of ES on pregnancy in women who underwent FET at Ebne-sina Infertility Center, Tehran, Iran, between January 2015 and December 2021. The study found that women who received ES were significantly older and had a slightly longer duration of infertility. No significant differences were observed in weight and BMI between the 2 groups. The distribution of causes of infertility was similar among the 2 groups. Regarding ET, the 2 groups were similar before and after the procedure. The rate of pregnancy and its outcomes (abortion and live birth) were not significantly different among the 2 groups. There were no reported complications during the in-center stay following the ES procedure.

The precise mechanism by which ES affects implantation remains unknown. One theory suggests that the mechanical injury caused by the procedure delays endometrial maturation, thereby synchronizing the embryo and receptive endometrium (3, 7). Additionally, the inflammatory response following ES can enhance the aggregation of immune cells and upregulate interleukin-15, tumor necrosis factor-α, and macrophage inflammatory protein-1B, potentially increasing implantation competency (8, 9).

Despite being introduced 2 decades ago, the efficacy of ES remains a topic of debate (6). A systematic review conducted in 2022 indicated that ES, specifically Pipelle biopsy, was associated with increased pregnancy (RR: 1.59, 95% CI: 1.24-2.03) and live birth rates (RR: 1.67, 95% CI: 1.26-2.21) (3). These findings align with previous meta-analyses, which demonstrated a notably higher pregnancy and live birth rate in IVF or intracytoplasmic sperm injection following pipeline biopsy or hysteroscopy, especially among cases with previous intrauterine insemination failure (10, 11). On the other hand, 2 recent systematic reviews suggested that ES (Pipelle biopsy or Novak curette) does not improve the pregnancy rates or outcomes (12, 13).

Furthermore, a meta-analysis of 10 randomized controlled trials demonstrated that the beneficial effects of ES on pregnancy and live birth rates were only evident for cases with multiple previous IVF failures. Also, it concluded that the timing and technique of ES play a crucial role in determining its effectiveness on embyo implantation (14).

The variability in systematic review conclusions may be due to differences in inclusion criteria, quality assessment methods, publication bias, and author expertise and bias. Moreover, these studies did not consider the nuanced differences in the interventions. Furthermore, original studies on ES reported different results and were also linked with significant risks of bias (12).

There are several reasons for the discrepancies observed in the trial studies (4, 15-18). Differences in the characteristics of the study population, the severity of the scratch, the phase of the menstrual cycle, and the appropriateness of the no-scratch group may contribute to these discrepancies. Some studies have performed ES on women who had experienced one or 2 IVF failures (16, 19), while some on the first cycle of IVF (17, 20, 21). Additionally, although ES is reported to be most effective in the luteal phase (22), some studies have not reported the phase of the menstrual cycle for ES (23).

Recently, a randomized controlled trial reported that endometrial injury (with Pipelle) does not significantly improve the outcomes of FET in cases with repeated implantation failures (24). Our study, which evaluated the efficacy of hysteroscopic scratch with scissors, has replicated these findings. Our findings in this matter align with other systematic review studies, indicating that ES does not lead to a higher incidence of pregnancy or live birth following FET (12, 13).

Recent reviews report that clinical trials involving ES are susceptible to high bias levels (12, 13, 25). Such biases and pitfalls can potentially lead to misleading conclusions (26). Additionally, most studies fail to report essential outcomes such as live births and adverse events. Furthermore, it is important to note that the infertility-affected population is a vulnerable group that may be willing to try anything to achieve pregnancy. It is crucial to thoroughly evaluate any adjuvant therapies before incorporating them into routine practice. By doing so, we can ensure that individuals receive the most effective and evidence-based treatments available.

### Limitations

Certain limitations arose from the retrospective nature of our study's design. Notably, the uneven distribution of older patients with extended infertility duration who received ES may have led to heterogeneity between the groups, potentially impacting our findings. Furthermore, due to the limited sample size, we were unable to conduct subgroup analyses pertaining to infertility causes, failed IVF attempts, and diverse baseline ET.

## 5. Conclusion

To our knowledge, this is the first study reporting the effects of endometrial scratch with scissors on the outcomes of FET. Endometrial scratching probably is not associated with a higher rate of pregnancy and live birth in FETs. Also, the number of previous embryo transfers and infertility duration would not affect the outcome of FET. Further, high-quality RCTs are recommended to address these issues.

##  Conflict of Interest

The authors declare no conflict of interest.
